# HCV cure without interferon

**DOI:** 10.1186/1471-2334-14-S2-S17

**Published:** 2014-05-23

**Authors:** Marc Bourlière

**Affiliations:** 1 Hepato-Gastroenterology Department, Saint Joseph Hospital, Marseille, 13285, France

## 

The launch of first-generation protease inhibitors (PIs) was a major step forward in HCV treatment. However, this major advance was restricted to genotype 1 (GT-1) patients. This year the launch of Sofosbuvir a NS5B nucleotide inhibitor (Nis), Daclatasvir a NS5A inhibitor (NS5A.I) and Simeprevir a second wave PIs open new perspective for IFN-free regimen. Both Sofosbuvir and Daclatasvir have a pan-genotypic activity. Sofosbuvir has highly potent antiviral activity across all genotypes in association with pegylated interferon and ribavirin (PR), thus allowing shortened treatment duration. Moreover, Sofosbuvir in combination with Daclatasvir without ribavirin for 12 weeks was able to cure > 95% of naïve GT-1 and for 24 weeks was able to cure 40 patients with treatment experienced, failure to triple therapy with first generation PIs, GT-1 patients. Sofosbuvir in combination with Simeprevir for 12 weeks without ribavirin was able to cure all GT-1 naïve patients and over 90% of treatment experienced GT-1 patients. As the three drugs have potent antiviral activities against GT-4, such association must be potent in such patient but data are pending.

For GT-2, Sofosbuvir and ribavirin for 12 weeks are able to cure > 90% of naïve and treatment-experienced patients. For GT-3 patients, Sofosbuvir and ribavirin for 24 weeks are able to cure around 90% of naïve and treatment experienced patients with the exception of treatment-experienced cirrhotic patients in whom SVR is around 60%. However the combination of Sofosbuvir and Daclatasvir for 24 week leads to more than 90% SVR in the naïve GT-3 population and trials are on-going for treatment-experienced patients. All this data confirmed that HCV can be cure with interferon–free regimen and short duration of treatment. Other combination with higher efficacy and very good safety profile are under development and should be available within two years.

